# Structural Correlates of Taste and Smell Loss in Encephalitis Disseminata

**DOI:** 10.1371/journal.pone.0019702

**Published:** 2011-05-17

**Authors:** Felix Alexander Schmidt, Onder Goktas, Lutz Harms, Georg Bohner, Katharina Erb, Bettina Dahlslett, Franca Fleiner

**Affiliations:** 1 Department of Otolaryngology - Head and Neck Surgery, University of Berlin, Charité Campus Mitte, Smell and Taste Consultation Service, Berlin, Germany; 2 Department of Neurology, Consultation Service for multiple sclerosis, University of Berlin, Charité Campus Mitte, Berlin, Germany; 3 Department of Radiology, University of Berlin, Charité Campus Mitte, Berlin, Germany; National Institutes of Health, United States of America

## Abstract

**Background:**

Olfactory dysfunction in MS patients is reported in the literature. MRI of the olfactory bulb (OB) is discussed as a promising new testing method for measuring olfactory function (OF).

Aim of this study was to explore reasons for and optimize the detection of olfactory dysfunction in MS patients with MRI.

**Materials and Methods:**

OB and olfactory brain volume was assessed within 34 MS patients by manual segmentation. Olfactory function was tested using the Threshold-Discrimination-Identification-Test (TDI), gustatory function was tested using Taste Strips (TST).

**Results:**

41% of the MS patients displayed olfactory dysfunction (8% of the control group), 16% displayed gustatory dysfunction (5% of the control group). There was a correlation between the OB volume and the number and volume of MS lesions in the olfactory brain. Olfactory brain volume correlated with the volume of lesions in the olfactory brain and the EDSS score. The TST score correlated with the number and volume of lesions in the olfactory brain.

**Conclusion:**

The correlation between a higher number and volume of MS lesions with a decreased OB and olfactory brain volume could help to explain olfactory dysfunction.

## Introduction

MS can be diagnosed using the McDonald criteria [1]. MRI is the imaging method of choice with inflammatory diseases of the CNS and the most sensible method in the diagnosis of MS [2]. MRI serves to depict acute as well as chronic MS lesions and their volume and allows physicians to measure the volume of additional regions in the brain. Olfactory disorders increasingly occur with Parkinson and Alzheimer's disease, but with less frequency with other neurodegenerative diseases [3], [4]. They often occur as early symptoms of these diseases. There are some scientific studies already that report olfactory disorders in MS patients at a rate of 15% [5], 22.5% [6] and 38.5% [7].

Our study wants to examine pathological tissue changes and volume changes of the olfactory brain and of the olfactory bulb (OB) of MS patients and correlate them with their olfactory and gustatory function. The aim is to investigate possible causes of the origin of olfactory disorders in MS patients in greater depth. For this purpose, volumetric measurements of the OB as well as the olfactory brain were performed as objective examination method for the first time. The volumes were determined with manual segmentation. The exact number, localisation and volume of the lesions were determined using a standardised MRI protocol specially developed for our study. Olfactory testing was performed using the tripartide Threshold-Discrimination-Identification-Test (TDI).

## Materials and Methods

The study was performed in the time from January 2009 until November 2009. Ethical approval and trial registration was obtained by the medical ethics comittee of Charité, University of Berlin. Written consent was required to participate in the study. 34 prospective patients (24 women, 10 men, 22–65 years, Ø 41 years, mean disease duration 6 years) were examined. 25 patients had relapsing-remitting MS, five patients primary progressive and four patients secondary progressive MS. The patients were included in resp. excluded from the study after an ENT and neurological examination as well as by completing two questionnaires. Patients with the diagnosis MS (McDonald criteria, revised version 2005) were included in the study. Exclusion criteria were: pregnancy, age below 18 or over 65, olfactory disorders with a different genesis (post-infectious, post-traumatic, sinunasal, infections of the upper respiratory tract, tumours treated with radiation or chemotherapy, allergies, patients suffering from depression, Parkinson's or Alzheimer's disease). Patients taking drugs that could cause olfactory dysfunction as for example methotrexat, amitryptilin, certain antibiotics and D-Penicillamine were excluded by the questionnaires. Furthermore patients receiving corticosteroid treatment up to six weeks before testing were excluded from the study because corticosteroids can have an effect on the OF [8]. To exclude dementia, the prospective patients were submitted to the Mini Mental State Examination (MMSE) [9]. A total score of at least 24 points was defined as exclusion criterion. Patients with grave physical disabilities were excluded using the Expanded Disability Status Scale (EDSS) [10]. A value of below seven was the threshold value for participation in the study. To exclude depression, a Becks Depression Inventory test (BDI) was performed. The BDI is a self-evaluation method for recording the severity of symptoms of depression [11]. MS patients show a higher depression level [12]. Disease-related symptoms, e.g. increased fatigue, lead to false higher scores in the BDI test. Adapted to the patients in our study, we defined a score of below 15 points as exclusion criterion. Patients with test scores of above 15 were tested once again by a psychologist using the Hamilton Rating Scale for Depression and the Hamilton Anxiety Rating Scale in an external assessment.

A complete ENT examination with endoscopy for exact anatomic evaluation of the nasal passage, the sinuses, damage to the nasal mucosa as well as the presence of polyps was performed to exclude olfactory disorders of another origin. Exemption criteria also applied for the participation in the MRI examination. The olfactory capacity was evaluated using the tripartide TDI test recommended by the “Working Group Olfactology and Gustology” of the German ENT Society (standardised, reliability r = 0.72) [13]. The Threshold test consists of 48 sniffing sticks with a 16-stage dilution series of n-butanol for determining the olfactory perception threshold of a patient. The discrimination test consists of 48 sniffing sticks to test the distinction of smells. Everyday smells have to be identified with the identification test. A TDI value of less than 16 means anosmia, up to 30 points hyposmia and above 30.5 points normosmia [14]. Paper taste test strips (TST) by Burghart, Wedel, Germany, were used to determine the sense of taste [15]. The 16-part test checks the four tastes sweet, sour, salty and bitter in four different concentrations each. The test strip is placed in the centre of the front third of the top of the tongue. Prior to each application, the mouth is rinsed with water. A test value of below 9 indicates a reduced sense of taste.

The patients were examined on a 1.5 Tesla MRI system (Symphony Vision, Siemens, Erlangen) using a standardised protocol to visualise the OB and the olfactory brain. The protocol contained 3 mm T2 turbo spin echo sequences (TR 3070 msec, TE 107 msec, Matrix 256×192, FOV 250 mm). 3 mm proton density sequences (TR 3070 msec, TE 18 msec, Matrix 256×192, FOV 250 mm) were applied to determine the MS lesion load by using axial sections. 3 mm T1-weighted sequences (TR 600 msec, TE 14 msec, Matrix 256×192, FOV 250 mm) were used for volumetric measurements of the olfactory brain.

Isotropic, 0.5 mm thick, high-resolution, strongly T2-weighed CISS sequences (constructive interference in steady state, TR 8.56 msec, TE 4.28 msec, Matrix 256×205, FOV 130 mm) were performed additionally for volumetry of the OB. Gadolinum was applied as contrast agent to allow a detailed assessment of the MS lesions.

The olfactory brain was defined as the piriform and entorhinal cortex, front agranular regions of the insular lobe up to the anterior comissure, orbitofrontal cortex. White matter in between these regions was also considered as part of the olfactory brain in order to take into account the fact that connections could have been damaged by MS lesions [16].

**Figure 1:**
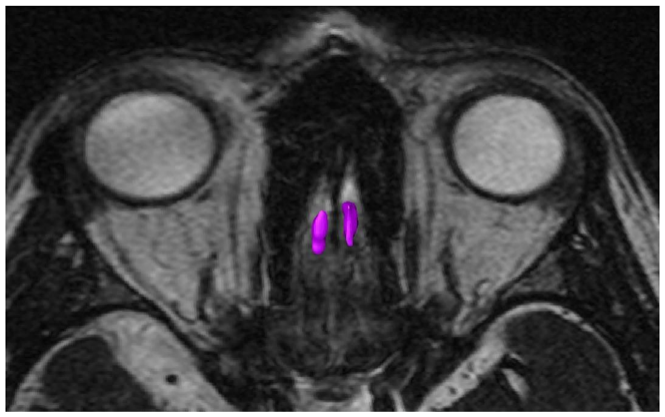
CISS sequence of OB, axial view.

The volume of the OB (Figure 1) and the olfactory brain was determined with MRI by circumnavigating the contours in axial sections with the computer software Amira 3.2.

We determined the number and volume of the lesions in the olfactory brain as well as in the rest of the brain. The examiners were blinded to the patient's olfactory test and EDSS scores. MRI measurements, olfactory and neurological testing have been performed on the same day.

Statistical analysis was performed with SPSS (17.0, Chicago, USA). Correlations were determined with a bivariate regression analysis. The results of the MRI evaluation were compared with smell and taste test values using the Pearson and Spearman correlation. The significance level was defined at ≤0.05.

The Welch test and the Brown-Forsythe test were used as statistic test method to check the equality of the mean values of different test parameters in patients with different types of progression. The Mann-Whitney U-test for independent samples was used to compare different control samples.

The patients were recruited continuously from the MS centre, Charité Campus Mitte. A healthy control group of 30 persons whose gender, age and smoking habits coincided with that of the patients were recruited among the patients' family members as well as among the hospital staff. All participants of the control group had to complete the same questionnaires and received an ENT examination in order to exclude the same causes for olfactory dysfunction as for the MS patients.

## Results

34 MS patients and 30 healthy control persons were examined. The volume of the OB (Table 1) of the 34 patients correlated with the number of MS lesions in the olfactory brain (r = −0.34, p<0.05) as well with the volume of the MS lesions in the olfactory brain (r = −0.37, p<0.05).

**Table 1:** Results of the MRI examination.

	Mean value	Standard deviation
Total volume OB	128.0 mm^3^	±41.54 mm^3^
Volume OB right	62.35 mm^3^	±21.08 mm^3^
Volume OB left	65.65 mm^3^	±22.89 mm^3^
Volume Olfactory Brain	38896.79 mm^3^	±7731.36 mm^3^
Number of lesions in the Olfactory Brain	2.38	±3.62
Volume lesions in the Olfactory Brain	90.37 mm^3^	±171.42 mm^3^
Number of lesions in the Non Olfactory Brain	43.35	±25.40
Volume of lesions in the Non Olfactory Brain	11599.0 mm^3^	±14333.61 mm^3^

The volume of the olfactory brain correlated with the volume of the lesions in the olfactory brain (r = −0.33, p = 0.05) and the EDSS value (r = −0.37, p<0.05). A significant difference of p = 0.03 was observed between the average volumes of the patient's olfactory brain with different types of progression of MS. The volumes of the patients with primary and secondary progressive form were lesser than those with relapsing-remitting progressive form.

Subjective olfactometry using sniffing sticks showed hyposmia in 41% of 34 MS patients (Table 2). In the healthy control group, 8% of the control persons showed hyposmia. 71% of MS patients with a decreased OB volume and 83% with a decreased olfactory brain volume displayed hyposmia. Nine of 34 patients refused the taste strip test. 16% of 25 patients had a gustatory dysfunction. In the control group, 5% of the patients had a taste disorder.

**Table 2:** Patient data: Results of olfactometry, gustometry and neurological examinations.

	Mean value	Standard deviation
Age (years)	41.4	±12.4
Duration of disease (years)	6.1	±7.9
TDI	31.5	±4.0
T (threshold)	6.3	±1.8
D (Discrimination)	12.4	±2.5
I (Identification)	12.8	±1.7
TST	10.8	±2.8
EDSS	3.3	±2.1
BDI	7.3	±6.4
MMSE	28.9	±1.1

The TST value correlated with the number of lesions in the olfactory brain (r = −0.49, p<0.05) as well as their volumes (r = −0.52, p<0.05).

The TDI value correlated with the EDSS score (r = −0.54, p<0.01).

## Discussion

The olfactory sense is of great importance in everyday life. The olfactory organ is a control function of the body and warns us about toxic substances and spoilt food. Some neurological disorders go hand in hand with olfactory disorders [3], [4]. Detecting olfactory disorders is becoming increasingly important in the research of neurodegenerative diseases [5].

There is a substantial need for diagnosis and the significance of imaging the olfactory system is also increasing continuously [17].

Special attention is paid to the OB, which seems to be an image of the neuronal afference of smelling due to its high plasticity [18]. A significant correlation between the OB volume and the olfactory ability was determined in several studies [19], [20], [21].

The olfactory ability is reduced with increasing OB hypoplasia [19], [20], [21]. An OB volume of <100 mm^3^ was determined as the cut-off value for a reduced OB volume [22].

In several studies, reduced OB volumes were determined in patients with olfactory disorders of a post-infectious, post-traumatic and sinunasal genesis [23], [24], [25]. A reduced OB volume was also observed in patients suffering from Alzheimer's disease and schizophrenia [26], [27]. The OB volumes were not reduced in patients suffering from Parkinson's disease [28].

In our study, we investigated the volume of the OB and the olfactory brain in MS patients and compared this to the number and volume of pathological lesions.

The OB volume of the 34 MS patients was diminished with a mean value of 128 mm^3^ compared to the standard values of the normal population with the same age (134 mm^3^) [21]. The correlation of number and volume of the plaques in the olfactory brain with the OB volume could explain the variability of the OB volumes in MS patients. The OB volume decreases with an increasing number and volume of MS lesions in the olfactory brain. These results may help to explain the occurrence of olfactory disorders in MS patients.

The olfactory brain volume correlated with the volume of MS lesions in the olfactory brain as well as the EDSS score. A reduced olfactory brain volume was detected in two studies with patients suffering from Parkinson's and schizophrenia [29], [30]. The larger the volume of the MS lesions in the olfactory brain, the more pronounced was the hypoplasia of the olfactory brain. 83% of patients with an olfactory brain volume of below 30000 mm^3^ displayed hyposmia. This correlation may also serve to explain olfactory disorders in MS patients. The olfactory brain volumes in MS patients with primary and secondary progression were significantly smaller than those of MS patients with relapsing-remitting progression. Demyelination with axon destruction results with chronic progredient progression, which might explain this significant difference in volume.

The olfactory brain volume as well as the TDI value correlated with the EDSS score, which provides information about the degree of disability of the MS patient. Several MS patients reported about a subjective improvement resp. deterioration of their olfactory sense in case of changed physical symptoms, e.g. during an acute episode. The TDI score seems to uncover changes in the olfactory capacity in MS patients. The TST values correlated with the number and volume of the lesions in the olfactory brain. On a cortical level, the gustatory and olfactory systems intersect, above all in the insular cortex, the amygdala and the orbitofrontal cortex. On downstream paths of the OB's mitral cells from the piriform to the orbitofrontal cortex, the olfactory stimuli are linked with gustatory information in the anterior insula (multimodal integration).

In our study, we investigated the volume of the OB and olfactory brain in MS patients for the first time and compared this to the number and volume of MS lesions. The correlation of a higher number and volume of MS lesions in the olfactory brain with a decreased OB and olfactory brain volume might explain the occurrence of olfactory dysfunction in MS patients. The results should be verified in a longitudinal study with a higher number of patients.
